# Improved HPLC Quantification of 6-Mercaptopurine Metabolites in Red Blood Cells: Monitoring Data and Literature Analysis

**DOI:** 10.3390/ijms231911885

**Published:** 2022-10-06

**Authors:** Tiphaine Adam de Beaumais, Yves Medard, Océane Amblard, Lauriane Goldwirt, Mathieu Simonin, Christine Martinez Vinson, Arnaud Petit, Evelyne Jacqz-Aigrain

**Affiliations:** 1 Department of Biological Pharmacology and Pharmacogenetics, Hôpital Saint-Louis, Assistance Publique—Hôpitaux de Paris, 1 rue Charles Vellefaux, 75011 Paris, France; 2 Department of Pediatric Hematology and Oncology, Hôpital Armand Trousseau, Assistance Publique—Hôpitaux de Paris, 26 Avenue du Docteur Arnold Netter, 75012 Paris, France; 3 Department of Education and Training, Sorbonne Université, 1 rue Victor Cousin, 75005 Paris, France; 4 Department of Pediatric Gastroenterology and Nutrition, Hôpital Robert Debré, Assistance Publique—Hôpitaux de Paris, 48 Boulevard Serurier, 75018 Paris, France; 5 Department of Education and Training, Université de Paris, 2 rue Albert Einstein, 75013 Paris, France

**Keywords:** thiopurines, 6-mercaptopurine, 6-thiopurines, monitoring, acute leukemia, chronic inflammatory bowel disease, pharmacogenetics

## Abstract

Thiopurine drugs azathioprine (AZA) and 6-mercaptopurine (6-MP) are used extensively in pediatric and adult patients with inflammatory and neoplastic diseases. They are metabolized to 6-thioguanine nucleotides (6-TGN) or to 6-methyl-mercaptopurine nucleotides (6-MMPN). The balance between 6-TGN and 6-MMPN is highly variable and monitoring is recommended, but its benefit in outcome gives rise to conflicting results, potentially increased by differences in quantifying 6-MP metabolism. Our aim was to report (1) the HPLC-UV procedure used in our laboratory to quantify red blood cells (RBCs) with 6-TGN and 6-MMPN (as its derivate: 6-MMP(d)) in patients treated with thiopurines and (2) additional tests, sometimes confirmatory, to improve method standardization. The comparison of two methods to count RBCs shows that metabolite concentrations were slightly lower in the washed and resuspended RBCs than in whole blood. Perchloric acid (0.7 M), dithiothreitol (DTT, final 0.013 M sample concentration) and 60 min hydrolysis were selected for acid hydrolysis. (3) Monitoring data from 83 patients receiving AZA or 6-MP showed that at steady state, only 53/183 (29%) had 6-TGN and 6-MMPN in the recommended therapeutic range. Our method is discussed in light of the technical conditions and sample stability data from 17 publications identified since the first analytical report in 1987. Monitoring data demonstrate, if required, that inter-patient variability in 6-TGN and 6-MMPN concentrations is high in samples from treated patients.

## 1. Introduction

Azathioprine (AZA), 6-mercaptopurine (6-MP) and thioguanine are thiopurine immunosuppressive drugs, primarily used in adults and children for the treatment of chronic inflammatory diseases [1,2,3,4] or onco-hematologic diseases [5,6,7,8,9,10] and also used in the past decades for the prevention or treatment of rejection during organ transplantation [11]. These drugs are highly metabolized, and their immunosuppressive and antiproliferative effects result from the incorporation of thiopurine metabolites into DNA, the inhibition of de novo purine synthesis and the modulation of RAC-1 activity [12,13,14].

**Figure 1:**
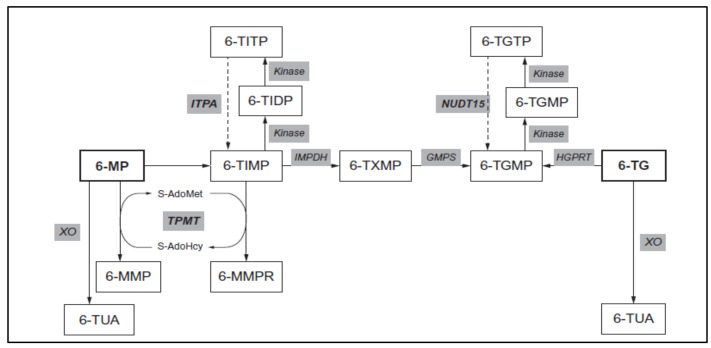
Detailed 6-mercaptopurine metabolic pathways presenting the enzymes involved. Compounds: 6-MP: 6-mercaptopurine, 6-TG: 6-thioguanine, 6-MMP: 6-methylmercaptopurine, 6-TUA: 6-thiouric acid, 6-MMPR: 6-methymecaptopurine ribonucleotides, 6-TIMP: 6-thioinosine monophosphate, 6-TIDP: 6-thioinosine diphosphate, 6-TITP: 6-thioinosine triphosphate, 6-TXMP: 6-thioxanthosine monophosphate, 6-TGMP: 6-thioguanine monophosphate, 6-TGDP: 6-thioguanine diphosphate, 6-TGTP: 6-thioguanine triphosphate, A-AdoHcy: S-Adenosyl-L-homocysteine, S-AdoMet: S-Adenosyl-L-Methionine. Enzymes: IMPDH: Inosine monophosphate dehydrogenase, Inosine monophosphate dehydrogenase, GMPS: Guanosine monophosphate synthetase, HGPRT: Hypoxanthine-guanine phosphoribosyltransferase, IMPDH: Inosine monophosphate dehydrogenase, ITPA: inosine triphosphate pyrophosphatase, NUDT15: Nudrix Type 15 - Nucleoside diphosphate linked moiety X--type motif 15 XO: Xanthine oxidase, TPMT: Thiopurine S- methyltransferase.

AZA is a prodrug, converted to 6-MP by reaction with reduced glutathione (GSH) in the presence of glutathione-S-transferase (GST), although some spontaneous conversion has also been reported [15,16,17]. 6-MP is a prodrug highly metabolized to 6-thiogunine nucleotides (6-TGN) and 6-methylmercaptopurine nucleotides (6-MMPN). Multiple pharmacogenetic interactions of thiopurine methyltransferase (TPMT) [18,19], inosine triphosphate pyrophosphatase (ITPA) [20] and Nudix hydrolase 15 (NUDT15) [21] affect 6-MP metabolism and “metabolite recycling”. Inter-subject variability in 6-TGN and 6-MMPN concentrations and their balance is known to influence 6-MP efficacy and toxicity—patients with high 6-TGN levels are at risk of myelosuppression, while patients with high levels of methylated metabolites are at hepatotoxic risk [22,23,24,25,26] (6-MP metabolism is detailed in Figure 1).

The multifactorial variability in the metabolism of these drugs supports therapeutic drug monitoring (TDM) of 6-TGN and 6-MMPN concentrations in patients treated with AZA and 6-MP (but not with thioguanine, as the measured concentrations result from the 6-TGN hydrolyzed fraction and from the drug itself). Monitoring is recommended, but its benefit in treatment outcome gives rise to conflicting results, potentially increased by differences in the procedure quantifying 6-MP metabolites in red blood cells (RBCs).

**Compounds:** 6-MP: 6-mercaptopurine, 6-TG: 6-thioguanine, 6-MMP: 6-methylmercaptopurine, 6-TUA: 6-thiouric acid, 6-MMPR: 6-methymecaptopurine ribonucleotides, 6-TIMP: 6-thioinosine monophosphate, 6-TIDP: 6-thioinosine diphosphate, 6-TITP: 6-thioinosine triphosphate, 6-TXMP: 6-thioxanthosine monophosphate, 6-TGMP: 6-thioguanine monophosphate, 6-TGDP: 6-thioguanine diphosphate, 6-TGTP: 6-thioguanine triphosphate, A-AdoHcy: S-adenosyl-L-homocysteine, S-AdoMet: S-adenosyl-L-methionine.

**Enzymes:** IMPDH: inosine monophosphate dehydrogenase, inosine monophosphate dehydrogenase, GMPS: guanosine monophosphate synthetase, HGPRT: hypoxanthine-guanine phosphoribosyltransferase, IMPDH: inosine monophosphate dehydrogenase, ITPA: inosine triphosphate pyrophosphatase, NUDT15: Nudix type-15 nucleoside diphosphate-linked moiety X-type motif 15, XO: xanthine oxidase, TPMT: thiopurine methyltransferase.

We report here the method used for many years in our laboratory to quantify 6-TGN and 6-MMPN concentrations. We also reviewed the published methods initially developed by Lennard [27] and by Dervieux and Boulieu [28,29]. The methods published since then were analyzed to identify significant technical modifications, with the aim to contribute to standardization of the analytical procedure [30].

## 2. Results

### 2.1. Standard Curves: Linearity, LOQ, Interferences

**Figure 2:**
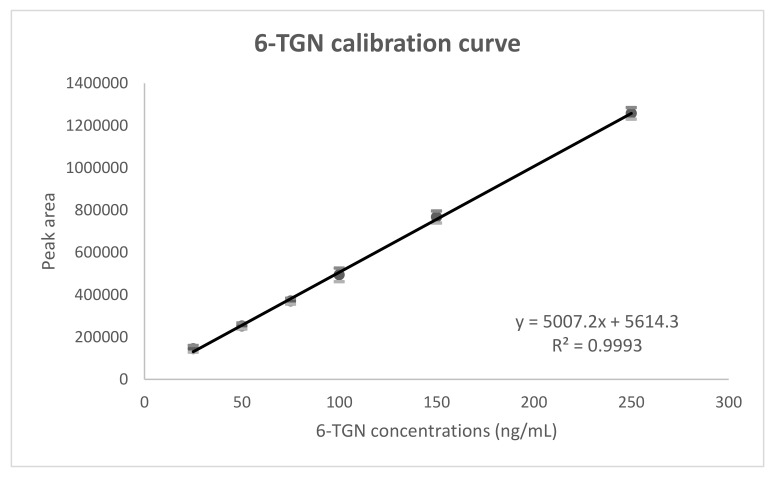

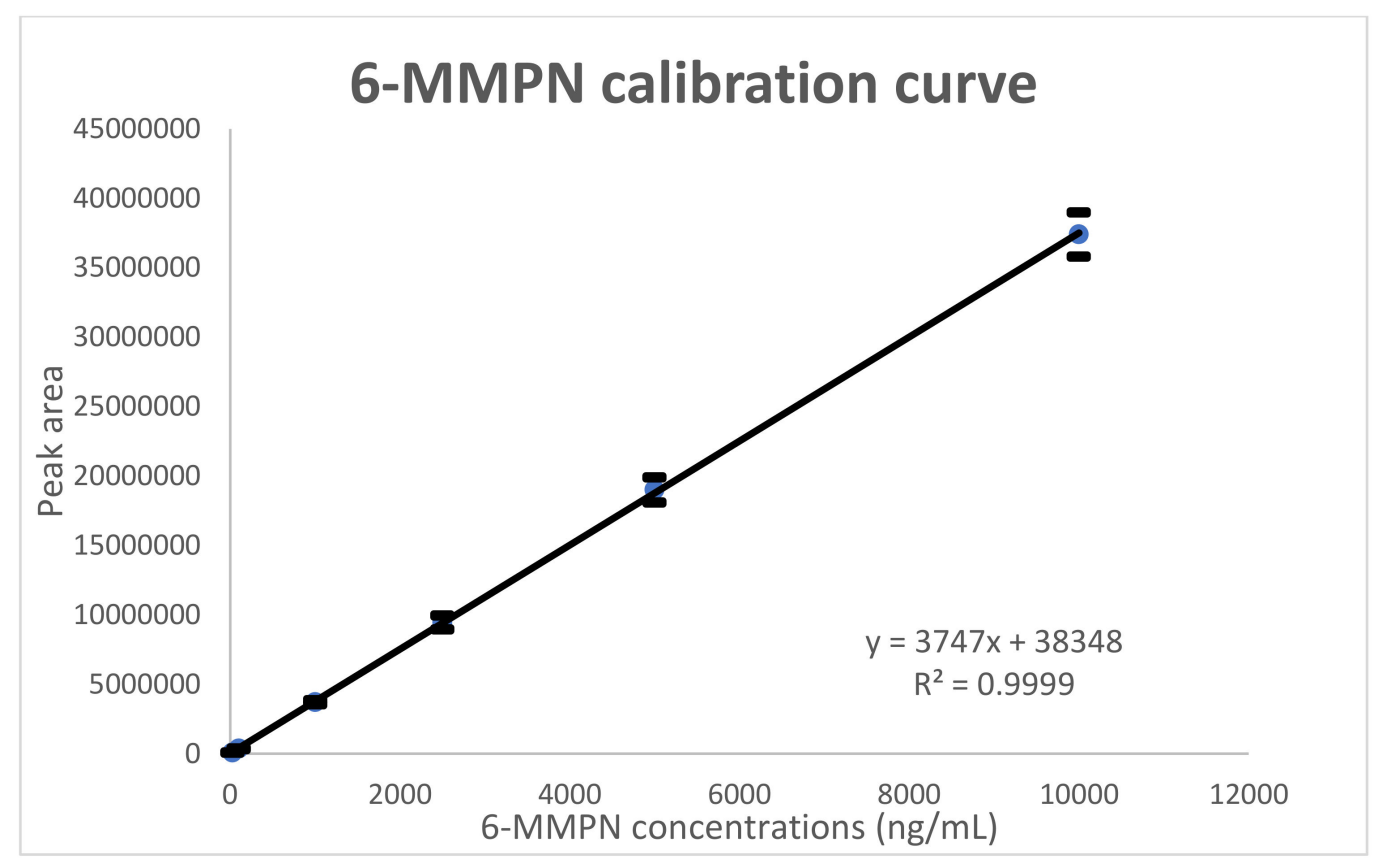
Mean calibration curves for 6-TGN and 6-MMPN.

The back concentrations of 6-TGN (25 to 250 ng/mL) and 6-MMP (25 to 10,000 ng/mL) were within 15% of the nominal values for the seven time points of the standard curves. The standard curves (*n* = 7) were linear with correlation coefficients over 0.995. For the seven replicates, the mean calibration curve was y = 5007.2x + 5614.3 (R² = 0.999) for 6-TGN and y = 3747x + 38348 (R² = 0.999) for 6-MMPN (Figure 2).

Mean (and standard deviation) concentrations (*n* = 7) are presented at each point.

Details of the seven calibration curves for both 6-TGN and 6-MMPN are also presented in Tables S1 and S2.

### 2.2. Selectivity

Blood blank samples did not show significant interferences from endogenous compounds at the retention times of 6-TGN and 6-MMP(d_4_) (*n* = 5).

### 2.3. Accuracy and Precision

Within-run (*n* = 5) and between-run *(n* = 7) quality controls were determined at concentrations of 40, 80 and 200 ng/mL for 6-TG and 500, 2500 and 7500 ng/mL for 6-TGN and 6-MMPN. The mean concentration of the three QCs of 6-TGN and 6-MMPN was within 15% of the corresponding nominal value in both cases, and coefficients of variation were below 10% for both 6-TGN and 6-MMPN. The low limit of quantification (LLOQ, *n* = 5) was fixed to 20 pmoL × 8 × 10^8^ RBC (Table S3).

### 2.4. Additional Tests Performed to Validate the Analytical Technique

Quantification of RBC count to express 6-TGN and 6-MMPN concentrations. RBC count was determined either in 100 µL packed RBCs after centrifugation and washing, or in whole blood immediately after sampling and without washing. The results were as follows: RBC count was 1.72 × 10^6^ ± 0.10 × 10^6^ RBC/µL in the packed RBC samples and 4.31 × 10^6^ ± 0.65 × 10^6^ RBC/µL in whole blood samples (n = 11, 6-TGN concentrations ranging from 50 to 285 pmol/8 × 10^8^ RBC). The final 6-TGN concentration was always higher when measured in washed and re-suspended RBCs (184 ± 73 versus 142 ± 56 pmol/8 × 10^8^ RBC, with a mean difference of 42 ± 18 pmol/8 × 10^8^ RBC between the two methods).

Impact of HClO_4_ final concentration. At the final concentration of 0.7 M, peak areas were higher at all standard concentrations. QCs for both compounds had accuracy over 90% (n = 3 for all QCs).

Impact of DTT concentrations on 6-TGN and 6-MMPN intracellular concentrations. Standard curves and quality controls were run in the presence of 65 µL of 0.2 M (reference method), 0.5 M and 1 M corresponding to final DTT concentrations of 0.013, 0.033 and 0.065 M, respectively. Back calculations showed that 6-TGN and 6-MMP concentrations and CQs were within 20% of the standard concentrations for all time points. The experiment also showed that the background noise increased with increasing DTT concentrations.

Duration of hydrolysis. Two durations of hydrolysis (60 and 90 min) were tested with samples from seven patients. 6-TGN and 6-MMPN concentrations were not different after 60 and 90 min hydrolysis.

Stability data (Table S4). QCs kept at −40 °C are included in all runs and were analyzed over 6 months (n = 49). Additional tests were performed to explore sample stability of (1) extracted QCs left at room temperature for 48 to 72 h, (2) washed and resuspended RBCs and subjected to three freeze/thaw cycles and (3) washed and resuspended RBCs kept at −20 °C for 15 days. The results showed limited impact on 6-TGN and 6-MMPN recovery.

When extracted, patients’ samples (n = 15) were re-run after being left at 4 °C for 48 h, and concentrations (1) decreased by 5.04% for 6-TGN (47.0 ± 8.6 and 44.6 ± 9.3 pmol/8 × 10^8^ RBC, with an absolute mean difference between samples of 3.9 ± 16.9 pmol/8 × 10^8^ RBC, n = 15) and (2) decreased by 1.38% for 6-MMPN (948.1 ± 1312.8 and 1135.0 ± 1300.7 pmol/8 × 10^8^ RBC, with an absolute mean difference between samples of 13.1 ± 14.3 pmol/8 × 10^8^ RBC).

## 3. Materials and Methods

### 3.1. Abbreviations

Red blood cells (RBCs), azathioprine (AZA), mercaptopurine (6-MP), 6-thioguanine (6-TG), 6-thioguanine nucleotides (6-TGN), 6-methylmercaptopurine (6-MMP), 6-methylmercaptopurine derivate ((6-MMP(d)): 4-amino-5-(methylthio) carboxy imidazole)), 6-methylmercaptopurine nucleotides (6-MMPN), dithiothreitol (DTT), perchloric acid (HClO_4_). An additional list of intermediate metabolites of 6-MP intracellular metabolism is included in Figure 1.

### 3.2. Reagents, Stock and Working Solutions

6-TG and 6-MMP were obtained from Sigma (59260, Lezennes, France). All chemicals were of analytical grade and purchased from Sigma. Stock solutions (1 mg/mL) of 6-TG (molecular weight, MW: 167.2) and 6-MMP (MW: 166.12) were accurately weighed and dissolved in methanol/NaOH containing 10 µL of 1 M DTT for 6-TG and methanol containing 10 µL DTT 1 M for 6-MMP.

Calibration curves, preceded by blank samples, were constructed for 6-TG (25, 50, 75, 100, 150, 250 ng/mL) and 6-MMP (25, 100, 1000, 2500, 5000, 10,000 ng/mL) by spiking known amounts of 6-TG and 6-MMP to washed erythrocytes obtained from our blood bank and controlled to eliminate interferences at the retention times of interest.

Quality control (QC) samples were prepared in packed RBCs to achieve final 6-TG and 6-MMP concentrations of 40/500, 80/2500 and 200/7500 ng/mL. Aliquots were prepared for standards and quality controls. All stock and working solutions were stored at −40°.

### 3.3. Sample Preparation

Blood samples to monitor thiopurines following prescription by physicians were obtained from patients treated with AZA or 6-MP, collected in heparinized tubes at 4 °C and centrifuged at 4000 rpm for 10 min at 4 °C to separate plasma from RBCs. RBCs were washed twice with 2 mL of 0.9% saline, packed by centrifugation and 100 µL was used to count the number of RBCs. The remaining packed erythrocytes were stored at −20°C. Patient monitoring was performed in agreement with the French law (Commission Informatique et Liberté no. 2226624); patients’ informed consent was not required.

Treatment of RBC Lysate. RBC hemolysate (100 µL) was homogenized with 65 µL DTT 0.2 M [27], 100 µL HClO_4_ 70% (0.7 M) and water in a final volume of 1000 µL. The mixture was vortex mixed for 30 s and centrifuged for 10 min at 4 °C. Acidic supernatant was transferred to a glass tube and heated at 100 °C for 60 min [27] to hydrolyze thiopurine nucleotides into their base. During this step, 6-MMP undergoes structural changes to the 4-amino-5-(methylthio) carboxy-imidazole (6-MMP(d)). After cooling, a 50 µL aliquot was injected into the HPLC system.

### 3.4. High Liquid Chromatography Analysis

The HPLC system consisted of a 1260 Infinity II LS system (Santa Clara, CA, USA). Chromatographic separation was performed on a C18 column Purospher RP18-e, 150 × 4.6 mm (Merck, Darmstadt, Germany). The mobile phase was a gradient of KH_2_PO_4_ 0.02 M (A) and CH_3_OH (B), starting at time 0 with 100% A, reaching 80/20 A/B at 12 min, 100/0 A/B at 15 min (100/0) and ending at 22 min, at a flow rate of 0.85 mL/min. Data were acquired with the Agilent OpenLab Chromatographic Data System—CDS (Santa Clara, CA, USA). The injection volume was 50 µL and the flow rate was 0.85 mL/min. Wavelengths were 342 nm (6-TG) and 304 nm (6-MMP(d)), and retention times were 6.4 min (6-TGN) and 10.7 min (6-MMP(d)). Typical chromatograms are presented in Figure S1.

### 3.5. Expression of 6-TGN and 6-MMPN Concentrations

Metabolite concentrations are expressed in pmol/8 × 10^8^ RBC according to the following equation: 5 x RBC count per mL × 10^6^ RBC/µL to take dilution into account. For comparison with packed RBC samples, the final metabolite concentrations based on whole blood count are also expressed in pmol/8 × 10^8^ RBC, after correction with the hematocrit value.

### 3.6. Validation Procedure

Calibration curves were established for 6-TGN and 6-MMPN on 7 different days. In the absence of an internal standard, the calibration curves were fitted by linear regression of the compound peak areas versus standard concentrations.

Accuracy and precision. Within- and between-run accuracy (determined/true value × 100) and precision ((standard deviation/mean) × 100)) were determined for QCs and for the lower limit of quantification. Coefficients of variations for the estimated concentrations and bias should not exceed ±20%.

Stability was tested with patients’ samples and 3 QC concentrations for 6-TGN (40, 80, 200 ng/mL) and 6-MMPN (500, 2500, 7000 ng/mL). In addition, tests initially performed at the concentrations of 40, 200 and 750 ng/mL for both 6-TGN and 6-MMP are reported.

Additional stability tests were performed (1) by keeping the extracted samples for 48 to 72 h at room temperature, (2) by subjecting washed and resuspended RBCs to 3 freeze/thaw cycles, (3) by keeping them at −20 °C for 15 days prior to analysis and (4) by keeping blood tubes for 48 h at +4 °C prior to any preanalytical treatment.

## 4. Monitoring of Treated Patients

**Figure 3:**
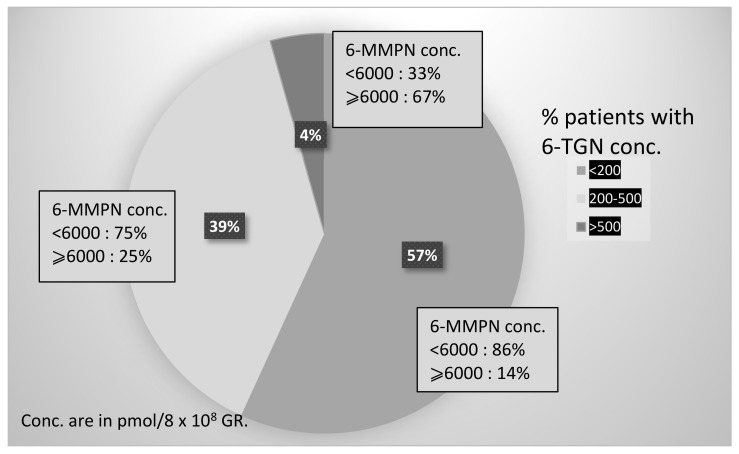
Percentage of patients with 6-TGN or 6-MMPN concentrations within or outside of the recommended therapeutic targets.

The first monitoring at steady state (at least 3 weeks after initiation of treatment, in the absence of transfusion) was performed in 183 patients treated with AZA or 6-MP. Patients’ characteristics are presented (Table S5). The target concentrations used were 200–500 pmol/8 × 10^8^ RBC for 6-TGN and lower that 6000 pmol/8 × 10^8^ RBC for 6-MMPN. Monitoring data show high variability in concentrations results, as 71/183 (39%) were in the therapeutic range for 6-TGN, and among them, 75% of patients were in the therapeutic range for 6-MMPN. A total of 130/183 patients (71%) were not in the therapeutic range, either for 6-TGN (112/183, 61%) or for 6-MMPN (38/183, 21%) or for both compounds (20/183, 11%) (Figure 3 and Table S5).

## 5. Discussion

Since their discovery by Elion G.B. and colleagues [31], thiopurine drugs have been used extensively in patients, both adults and children, to treat inflammatory and neoplastic diseases. These drugs are highly metabolized by multiple enzymatic steps to 6-MP nucleotide metabolites. The balance between 6-MP metabolite pathways is highly variable between diseases and pharmacogenetic polymorphisms affecting TMPT [18,19], ITPA [20] and NUDT15 [21] and is known to influence both efficacy and side effects—patients with high 6-TGN levels are at risk of myelosuppression, while patients with high levels of 6-MMPN derivates are at hepatotoxic risk.

Monitoring of thiopurines remains a matter of debate in treated patients [32,33,34,35,36], as thiopurine metabolite concentrations in RBCs are surrogates to the intracellular levels of these metabolites in the target tissues [37,38]. In addition, there is a need for standardization and external quality assessment for the pre-analytical and analytical methods [30,39,40].

We briefly report here the HPLC method used for many years in our department and review the HPLC-UV methods published since the first ones by Lennard [27] and Boulieu [28]. Our aim was to summarize and discuss the differences in the procedures that might contribute to conflicting data. In the present work, we focused on reported HPLC-UV methods as they are adapted to routine monitoring, although HPLC-MS and tandem MS methods, more complex and time-consuming, are also used, as well as some separate ribonucleotide mono-, di- and triphosphates [41,42,43,44,45,46,47].

In the first HPLC-UV methods by Lennard [27,48], also used by Erdman [49] and Pike [38], blood was collected on an anticoagulant (either lithium heparin or EDTA), the sample was centrifuged and RBCs were washed and resuspended, followed by deproteinization and acid hydrolysis at a high temperature with the formation of toxic phenylmercuric acetate into toluene and with sulfuric acid for the conversion of thiopurine nucleotides into their free bases. Due to the toxicity of the procedures, these first methods were rapidly modified and used perchloric acid in the presence of DTT [28,50]. This resulted in increased extraction efficiency from 40% to 84% [36], probably related to more complete protein precipitation and higher hydrolysis of TG phosphate groups [41,51]. During acidic hydrolysis at high temperatures, 6-MMP undergoes conversion to an amino-carbonyl imidazole derivate (6-MMP(d)), and this conversion, strongly influenced by the pH of the acid extract, is complete at pH = 0 and only partial in milder hydrolysis conditions [38].

**Figure 4:**
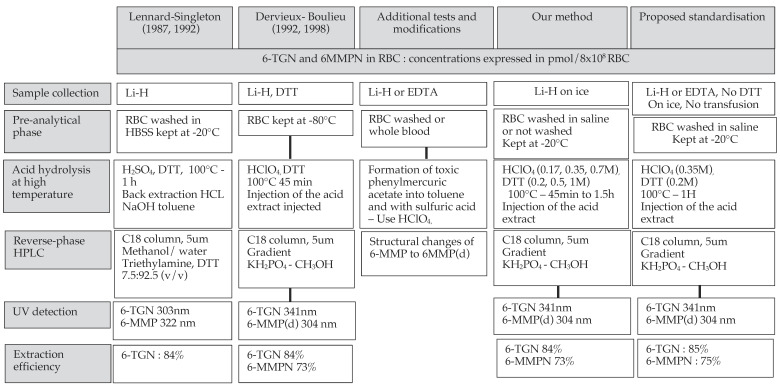
Summary of the first pre-analytical and analytical procedures by Lennard and Singleton and by Dervieux and Boulieu [27,28,29], the major modifications (presented in detail in Table S6) and a proposed standardized procedure based on additional tests.

According to our review (Figure 4, Tables S6 and S7)

1. It is recommended to collect samples on ice in tubes containing either heparin or EDTA, count RBCs either in whole blood or after washing and store packed RBCs at −20 °C [52,53].
2. 6-TGN and 6-MMPN concentrations are usually expressed in pmol/8 × 10^8^ RBC. We observed that 6-TGN concentrations were higher in washed and packed RBCs—probably related to the precision of the pipetting process - than in whole blood, as the contribution from white blood cells to the final metabolite concentrations is limited. Less frequently, hematocrit or hemoglobin are used to standardize and express 6-MP metabolite concentrations, as this contributes to overcoming the impact of anemia [43,54]. However, whole blood HPLC techniques require sulfuric acid to suppress interferences at TGN retention time [38].
3. In our method, the concentration ranges for the standard curves are set to 25 to 250 ng/m for 6-TGN and 25 to 10,000 ng/mL for 6-MMPN, allowing us to quantify concentrations in almost all treated patients
4. Perchloric acid final concentrations of 0.175, 0.35 and 0.7 M had no impact on 6-TGN concentrations, but 6-MMPN recovery was lower at the lowest concentration, and the concentration of at least 0.35 M used by many authors is recommended. Sulfuric acid is not recommended.
5. DTT, added during extraction to prevent binding of thiopurines to the acid denatured proteins of RBCs [55], influenced 6-TGN but did not influence 6-MMPN recovery [22]. DTT may also be added to the sample tube and/or the mobile phase, as proposed in different publications. Final DTT concentrations used during sample preparation and calculated from the information provided in the corresponding publications were extremely variable (ST6), but we confirmed that the lowest final concentrations of DTT (0.013 M), used in most published procedures, had no impact on 6-TGN and 6-MMPN recovery.
6. We also confirmed that increasing the duration of hydrolysis from 60 to 90 min does not increase 6-TGN recovery.

The published sample stability information during the pre-analytical and analytical phases is summarized in Table S3. Whole blood samples have limited stability at room temperature and should be collected on ice and kept at 4 °C for a few hours before being conditioned and extracted. Extracted samples can be kept for weeks, even months, at −20 °C or −80 °C without reductions in 6-TGN and 6-MMPN recovery. Additional stability tests are summarized in Table S7).

In addition to conflicting data on the individual benefit of monitoring thiopurines and the need to standardize the analytical methods, the interpretation of monitoring requires agreement on the optimal target concentrations for both 6-TGN and 6-MMP. Thresholds are reported for both compounds regardless of the drug (AZA or 6-MP), the disease and patient age. The target values of RBC 6-TGN levels between 230 and 450 pmol/8 × 10^8^ RBC are associated with a therapeutic response, while higher concentrations risk toxicity and lower concentrations risk ineffectiveness both in adults and children with Crohn’s disease or leukemia [1,30,32,55,56]. In the literature, a target 6-MMPN threshold of 5700 pmol/8 × 10^8^ RBC was defined for patients treated for inflammatory bowel disease, and 6-MMPN levels above 5700 increase the risk of hepatotoxicity and myelotoxicity [32]. We also showed that a 6-MMP threshold of 4884 pmol/8 × 10^8^ RBC was predictive of hepatotoxic risk in all pediatric patients during maintenance therapy [24].

In our experience, the results of monitoring thiopurines showed that only 53/183 patients (29%) had both 6-TGN and 6-MMP concentrations at steady state in the recommended therapeutic ranges, demonstrating that variability is high, if needed, and that observance is an important issue. The impact of monitoring on dosage adaptation and patient benefit is currently under analysis.

In conclusion, our pre-analytical and analytical HPLC-UV conditions (0.7 M perchloric acid, 0.013 M DTT and 1 h hydrolysis) to measure 6-TGN and 6-MMP was developed with the information already available in the literature since the initial reports [27,28,29] and would allow standardization of the key steps of the analytical procedure quantifying 6-TGN and 6-MMPN by HPLC in washed and resuspended RBCs or in whole blood. Wide ranges of 6-TGN and 6-MMPN were measured, demonstrating, if needed, that variability is high.

## Data Availability

Data are available on request due to privacy/ethical restrictions.

## Supplementary Materials

The following supporting information can be downloaded at: https://www.mdpi.com/article/10.3390/ijms231911885/s1, Table S1: Details of the calibration curves *(n* = 7) for 6-TGN and 6-MMPN quantification in red blood cells. Table S2: Back-calculated concentrations obtained from the 7 calibration curves and pooled standard curves for 6-TGN and 6-MMP. (**A**) Back-calculated concentrations are presented for 6-TGN (A) and 6-MMPN (B). (**B**) Mean and standard deviation concentrations at the different concentration-points are presented for the 7 standard curves and for the pooled standard curve. Table S3: Accuracy and precision of 6-TGN and 6-MMP quality controls. (**A**) Within- and between-run accuracy and precision determined for 3 QCs of 6-TGN and 6-MMP and for the lower limit of quantification. (**B**) Accuracy and precision determined for 3 QCs kept at −40 °C and used over 6 months. Table S4: Stability data. (**A**) Stability data of 6-TGN and 6-MMP QCs in 3 different conditions. (**B**) Stability of QCs kept up to 6 months at −40 °C and unfrozen for 6-TGN and 6-MMP sample quantification. Table S5: 6-TGN and 6-MMPN concentrations measured in patients treated with azathioprine or 6-mercaptopurine. Table S6: Summary of published pre-analytical and analytical methods of 6-TGN and 6-MMPN quantification in red blood cells. Table S7: Review of sample stability data during pre-analytical and analytical methods to quantify 6-TGN and 6-MMPN in red blood cells. Figure S1: HPLC typical chromatograms showing 6-TGN and 6-MMP(d) peaks at 342 and 304 nm.
