# Supplementary material for: Improved HPLC Quantification of 6-Mercaptopurine Metabolites in Red Blood Cells: Monitoring Data and Literature Analysis
Source: Int J Mol Sci. 2022 Oct 6;23(19):11885. doi: 10.3390/ijms231911885 (PMC9570176; doi:10.3390/ijms231911885)
Supplement: Supplementary file 1 [file ijms-23-11885-s001.zip › ijms-1908197-supplementary.pdf]

Table S1 Details of the calibration curves (n=7) for 6-TGN and 6-MMPN quantification in red blood cells.

6-TGN calibration curves (n=7)

CC1	y = 4908x - 1870	R^2 = 0.9966
CC2	y = 4997x + 813,9	R^2 = 0.9919
CC3	y = 4786.2x + 15772	R^2 = 0.9995
CC4	y = 5009.4x + 10352	R^2 = 0.9996
CC5	y = 5053.8x + 5239,5	R^2 = 0.9991
CC6	y= 5141.4x + 8840	R^2 = 0.9964
CC7	y = 5154.7x + 153,31	R^2 = 0.9998
**POOL**	**y= 5007.2x + 5614.3**	**R^2 = 0.9993**

6-MMP calibration curves (n=7)

CC1	y = 3999.8x + 17525	R^2 =1
CC2	y = 3586.8x + 20762	R^2 = 0.9997
CC3	y = 3563.5x + 85633	R^2 = 1
CC4	y = 3563.5x + 85633	R^2 = 1

CC5	y = 3675.5x + 60420	R^2=1
CC6	y = 3563.5x + 85633	R^2 = 1
	y = 3906x + 107997	R^2 =
CC7	0.9992	
	**y = 3747x + 38348**	**R^2 =**
**POOL**	**0.9999**	

Table S2

Back calculated concentrations obtained from the 7 and pooled standard curves for 6-TGN and 6-MMPN

Back calculated concentrations are presented for 6-TGN (A) and 6-MMPN (B) respectively.

Mean and standard deviation values for concentrations at the different standard points are presented for the 7 standard curves and for the pooled standard curve

**A. 6-TGN nominal values (ng/mL)**	25	50	75	100	150	250
Accepted range of back concentrations (15%)	21.25-28.75	42.5-57.5	63.75-86.25	86-115	127.5-172.5	212.5-287.5
Mean (n=7)	27.63	48.95	72.30	96.81	151	248
SD	10.04	2.24	3.89	4.20	2.38	7.15

Pooled standard curve	24.21	43.03	72.82	97.66	152.26	250.08

**B. 6-MMPN nominal values (ng/mL)**	25	100	1000	2500	5000	10000
Accepted range of back concentrations (15%)	21.25-28.75-	86-115	860-1150	2125-2875	430-575	8600-11500
Mean (n=7)	12.16	87.13	984.08	2567.5	5049.6	9975.2
SD	10.3	10.6	46.8	153.0	283.6	514.6
Pooled standard curve	21.5	95.5	989.5	2609.3	5071.8	9983.0

6-TGN: 6-thiogluanine nucleotides, 6-MMPN: 6-methylmercaptopurine nucleotide, SD: standard deviation

Table S3

Within and between run accuracy and precision determined for 3 QCs of 6-TGN and 6-MMPN and for the lower limit of quantification.

A) Accuracy and precision determined for 3 QCs kept at -40°C and used over 6 months

**A**	**6-TGN**	**6-MMPN**
**Intra-day (n=5)**		
Back calculation		
QC1	37.6 ± 2.0	458.9 ± 24.2
QC2	78.0 ± 2.4	2478.7 ± 33.4
QC3	204.3 ± 2.7	7041.2 ± 84.9
Accuracy		
QC1	93.8 ± 5.0	91.8 ± 4.8
QC2	97.4 ± 3.0	99.1 ± 1.3
QC3	102 .1 ± 1.3	93.9 ± 1.1
Precision		
QC1	5.4	5.2
QC2	3.1	1.3
QC3	1.3	1.2
**Interday (n=7)**		
Back calculation		
QC1	41.27 ± 1.2	496.50 ± 7.6
QC2	79.32 ± 3.4	2506.03 ± 107.1
QC3	196.64 ± 8.3	6868.19 ± 355.7
Accuracy (%)		
QC1	103 ± 3	99 ± 2
QC2	98 ± 4	100 ± 4
QC3	98 ± 4	98 ± 5
Precision (%)		
QC1	2.8	1.5
QC2	4.0	4.2
QC3	4.2	5.2
**LOQ (n=5)**		
5 ng/mL<br>Expressed in<br>pmoLx 8.10^{8} GR*	1.6 ± 0.3<br>30	1.1 ± 0.3<br>30
7 ng/mL<br>Expressed in<br>pmoLx8.10^{8} GR*	8.2 ± 0.2<br>42	1..0 ± 0.5<br>42
10 ng/mL<br>Expressed in	11.0 ± 0.3<br>60	12.3 ± 0.9<br>60

pmoLx 8.10^8 GR*		

Accuracy is the ratio of determined/true value *100

Precision is the ratio of the standard deviation/mean *100

QCs for 6-TGN : 40, 80, 200 ng/mL QCs for 6-MMPN : 500, 2500, 7000 ng/mL.

*: 6-TGN and 6-MMPN concentrations used the "washed RDC count" of 1.6x10^6 RBC

Table S4

A. Stability data of 6-TGN and 6-MMPN QCs in 3 different conditions

QCs	Nominal value (ng/mL)	6-TGN (ng/mL)				6-MMPN (ng/mL)		
		Mean concentration ng/mL, n=3	SD	Recovery (%)		Mean concentration ng/mL, n=3	SD	Recovery (%)
**Extracted samples kept 48-72h at room temperature**								
QC	40	36.8	1.3	92		39.9	3.6	99.6
QC	200	170.8	11.4	85		193.2	12.3	96.6
QC	750	689.8	25.4	92		716.4	2.0	95.5
**3 freeze / thaw cycles**								
QC	40	45.4	1.0	114		41.8	3.5	104.4
QC	200	199.7	1.8	100		195.4	5.5	97.7
QC	750	733.9	5.2	98		736.6	0.9	98.2
**Washed RDB maintained at 20°C for 15 days**								
QC	40	36.5	1.3	91		44.6	4.5	111.4
QC	200	171.4	26.6	86		196.0	8.1	98.8
QC	750	703.3	38.2	94		745.5	7.8	99.4

Recovery % = (practical concentration /theoretical concentration) x100%

B. Stability of QCs kept up to 6 months at -40°C and unfrozen for 6-TGN and 6-MMPN sample quantification

	**QC1 (n=49)**		**QC2 (n=49)**		**QC3 (n=49)**	
Thiopurines metabolites	6-TGN	6-MMP	6-TGN	6-MMP	6-TGN	6-MMP
**Nominal values (ng/mL)**	40	500	80	2500	200	7000
**Mean (ng/mL)**	40. 31	504.37	79.58	2511.03	195.03	6853.13
**Standard deviation (ng/mL)**	1.33	1366	2.75	93.80	5.65	222.70
**Biais**	0.77	0.87	0.55	0.44	2.48	2.1
**Coefficient of variation (%)**	3.31	2.71	3.46	3.74	2.9	3.25

Table S5

6-TGN and 6-MMPN concentrations measured in patients treated with azathioprine or 6-mercaptopurine

**A. Characteristics of the patients (n=183)**	
Age (mean ± standard deviation (range) (	29 ± 20.47 (0.8-85.1)
Gender (M/F)	78/105
Disease :<br>▪ Inflammatory bowel disease<br>▪ Hematological disease<br>▪ others	 118 (64 %)<br>40 (22 %)<br>25 (14 %)
Indication of monitoring :<br>▪ Systematic monitoring<br>▪ Insufficient response<br>▪ Doubt on compliance<br>▪ Adverse event	 118 (64 %)<br>34 (19 %)<br>20 (11 %)<br>17 (9 %)
6-TGN concentrations (pmol/8*10^8GR)	210 ± 131 (20-768)
6-MMPN concentrations (pmol/8*10^8GR)	3434 ± 5673<br>948 (20-40970)
Metabolic ratio 6-MMP/6-TGN	17.72 ± 31.18 (0.20-282.81)

**B. 6-TGN and 6-MMPN concentrations according to the corresponding therapeutic targets**		
	**Concentrations 6-TGN (pmol/8*10^8GR)**<br>Median (range)	**Concentrations 6-MMPN (pmol/8*10^8GR)**<br>Median (range)
**6-TGN therapeutic range**		
**200 < 6-TGN < 500 (n=71)**	**270 (203-499)**	**1334 (90-22550)**
6-MMPN <6000 (n=53)	263 (203-499)	619 (90-5618)
6-MMPN >6000 (n=18)	294 (209-390)	8407 (6195-22550)
**6-TGN below therapeutic range**		
**6-TGN < 200 (n=104)**	**130 (20-199)**	**709 (20-26768)**
6-MMPN <6000 (n=89)	124 (20-199)	570 (20-5084)
6-MMPN >6000 (n=15)	152 (66-199)	8991 (6817-26768)

**6-TGN above therapeutic range**		
**6-TGN > 500 (n=8)**	**591 (530-768)**	**6409 (206-40970)**
6-MMPN <6000 (n=3)	542 (530-616)	495 (206-2335)
6-MMPN >6000 (n=5)	599 (564-768)	12649 (6381-40970)

Table S6 : Summary of published pre-analytical and analytical methods for 6-TGN and 6-MMPN quantification in red blood cells

	RBC Count	1) Sample preparation<br>2) Extraction<br>3) Calibration curves	Chromatography /detection<br>Extraction efficiency	Comments
Lennard 1987 [27] 1992 [40]	Li-H tubes<br>WP RBC (in HBSS)<br>Kept at -20°C	**1) Final volume : 1500 µL**<br>200 µL RBCs,<br>800 µL DTT 3.75 mM (2mM*)<br>500 µL H2SO4 (1.5M)<br>1h –100°C<br>2)Extraction NaOH (500 µL, 3.4M), toluene, amyl alcool-PMA, HCL (200 µL , 0.1M)<br>2) 6-TG: 0.03-0.9<br>6-MMP:0.3-30 nmolx8.10^8 RBC	Methanol/water + thiethyamine + DTT, pH : 3.2<br>Reverse phase HPLC – UV<br>Injection volume : 50 **µL**<br>Extraction efficiency<br>6-TGN: 64 - 68 %<br>6-TIA :73%	Results in pmolx8.10^8 RBC<br>Additional thiopurines, including 6-TIA are quantified<br>Extraction efficiency is influenced by pH of adduct
Bruunshuus Schmiegelow 1989 [54]	Li-H tubes<br>RBC in SDS<br>DTT	**1) Final volume : 1000 µL**<br>50 µL RBC (+ water and SDS)<br>2) <u>Extraction</u><br>6 mg mercuricellulose	HPLC - UV<br>Isocratic elution buffer (KH_2PO_4/H_3PO_4)<br>Injection volume : 100 **µL**	Results in nmol/mmol hemoglobin<br>Additional compounds : 6MP, 6TU

		400 µL mercaptoethanol (60mM)<br>100 µL H_2SO_4 (2.5M)<br>1h - 98°C<br>3)6-TGN :250-6000 nmol/L	Extraction efficiency TGN : 40%	
Erdman 1990 [49]	WP RBC in saline	<u>6-TGN – MP assay</u><br>**1) Final volume : 100 µL RBCs**<br>100 µL (TG + MP)<br>200 µL Sulfuric acid (1M)<br>45min - 100°C<br>**2)** Extraction 250 µL + 10 µL 50% HClO4, dichloromethane, sodium bicarbonate (pH10), Potassium permanganate.<br>**3)** 6-TGN : 5-500 ng, IS SM/X<br><u>6-MMP, MTG assay</u> : sample preparation followed extraction with ethyl-acetate	<u>TG, MP</u> : Methanol / sodium phosphate + tetrabutylamonium chloride, pH : 7.5, fluorescence detection<br>Injection volume : 25 **µL**<br><u>MMPN, MTG</u>: Methanol / sodium phosphate,<br>pH: 2.7<br>HPLC- UV detection<br>Extraction efficiency :<br>6-TGN : 74-81%<br>6-MMP: 38-41%	Results in ngx8.10^8 RBC<br><br>Determination of TG, MP, MMP and MTG concentrations<br><br>No DTT
Boulieu Lenoir 1993 [50]	Sample Hi-L+ DTT	**1)Final volume : 1100 µL**<br>1 mL RBC<br>100 µL Perchloric acid 70%<br>10 mg DTT (58 mM*)<br>45 min 100°C<br><br>2)Linearity for 6-TG up to 7500 ng/mL	Reverse phase HPLC – UV<br>Elution gradient (KH_2PO_4 / KH_2PO + methanol<br>Injection volume : 60 **µL**<br><br>Extraction efficiency 81% if DTT in sample tube and treatment<br>50% if no DTT during deproteinisation)	- Separation of thiopurine bases, nucleosides, nucleotides<br>- Chromatographic retention of the thiopurine bases is not affected by pH (3 to 7) of the mobile phase but by methanol concentration.

				- DTT increases recovery of thiopurine bases
Dervieux Boulieu 1998 [28]	Sample Hi-L + DTT	**Final volume : 550 µL**<br>500 µLRBC<br>5mg DTT (58mM*)<br>50 µL perchloric acid 70%<br>45min 100°C<br><br>3)6-TGN: 0.3 – 50 µmol/L<br>6-MMP: 1.5 – 120 µmol/L<br>IS : 5-BU	Reverse phase HPLC – UV<br>Elution gradient (Potassium phosphate / methanol)<br>Injection volume: 80 **µL**<br><br>Extraction efficiency<br>6-TGN :73%<br>6-MMP : 84%	Results in pmolx8.10^8 RBC<br><br>6-MMP (d) formed at pH=0
Mawatari 1998 [47]	Sample Hi-L + DTT<br>WP RBC	**1)Final volume : 1100 µL**<br>1 ml RBC<br>100 µL Perchloric acid 70%<br>10 mg DTT (58 mM*)<br>Heating (no precision)<br><br>3)6-TGN : 20-200 pmol / 25 mg Hb<br>6-MMP : 18-1800 pmol /25 mg Hb	Reverse phase HPLC-UV<br>Elution gradient (methanol/phosphate buffer heptanesulfonic -acid sodium salt	Results in pmol /25mg Hb<br>Linear relationship between heated and non-heated 6-MMPN concentrations.
Shipkova 2003 [51]	Differences in the RBCs 6-TGN concentrations, attributed to duration of hydrolysis, acidification, DTT concentration, duration of hydrolysis ….			
Stefan 2004 [41]	EDTA<br>WP RBC	*Modification of Lennard Singleton (1992)*<br>**1)Final volume 1750 µL**<br>400 µL RBCS<br>800 µL DTT 3.75mM (2.1mM*)	Reverse phase HPLC – UV<br>Isocratic elution<br>Acetonitrile/phosphate buffer (pH2.5)	Results in pmolx8.10^8<br><br>6-TG / 6-MMP solubility higher in NaOH 0.1M

		25 µL IS<br>500 µL sulfuric acid 0.5M<br>75 min - 100°C<br>2)Extraction NaOH, toluene, amyl alcool, PMA / HCL<br>Compared to<br>*Modifications of Dervieux 1998*<br>Final volume 525 µL<br>400 µL RBCS<br>50 µL DTT 0.5M (47mM*)<br>25 µL IS<br>50 µL $HClO_4$ 0.5M<br>45 min - 100°C<br><br>3) 6-TGN :100 – 800<br>6-MMP: 750 – 6000<br>pmolx8.10^8 RBC<br>IS : 6-MP	Extraction efficiency : 6TG : recovery higher with alcalin pH	Extraction of 6TG is pH dependent
De Graaf 2010 [53]<br><br>adapted from Shipkova [51]	Li-H<br>WP RBC Resus-pended in PBS	1) **Final volume 365 µL**<br>250 µL RBCs<br>45 µL $HClO_4$ 70%:<br>20 µL DTT 1.1M (60mM*)<br>100°C , 1h<br>3) 6-TGN : 300 – 3600<br>6-MMP:1200-15000 pmolx8.10^8 RBC<br>IS : 5-BU	Reverse phase HPLC - UV – C18<br>Elution gradient: ACN in potassium dihydrogen-phosphate pH3.5/ACN<br><br>Injection volumr : 50 **µL**	Results in pmolx8.10^8 RBC<br><br>Used to test stability of thiopurine metabolites

Present report	EDTA<br>WP RBC	**1) Final volume : 1015 µL**<br>100 µL RBCS<br>65µL DTT 0.2M (13mM)<br>100 µL $HClO_4$ (70%)<br>750 µL H2O<br>2) 6-TGN : 25-250 ng/mL<br>6-MMP: 25-10000 ng/mL	Reverse phase HPLC - UV – C18<br>C18 column,<br>Elution gradient: KH_2PO_4 0.02M (A) and CH_3OH (B),<br>Wavelength is 342 nm (6-TGN) and 304 nm (6-MMP(d))<br>RT 6.4 min (6-TG) and 10.7 min (6-MMP(d))	Results in pmolx8.10^8 RBC
Dervieux 2005 [42]	EDTA<br>WP RBC<br>Stored at -70°C	Sample treatment according to Dervieux 1998<br><br>**1) Final volume 450 µL**<br>100 µL RBCS<br>300 µL DTT 0.2M (133mM*)<br>10 µm/L 8-BA<br>40 µL perchloric acid 70%<br>100°C , 1h<br><br>3) 6-TGN: 0.25 – 20<br>6-MMP: 2.5 -200 µmol/L<br>IS : bromoadenine	LC-MSMS<br><br>C18Isocratic elution<br>95% amonium acetate - formic acid 5% acetonitrile -formic acid.<br><br>6-TG : m/z 168-151<br>6-MMP : m/z 158-110	6-TGN/6-MMPN concentrations 17% / 18% higher compared to the methylmercury method by Lennard and Singleton 1992 and Cuffari<br>Relation with TMPT genotypes in treated patients
Kirchlerr 2013 [43]	Li-H / EDTA<br>WP RBC	**1) Final volume 395 µL**<br>25 µL RBC<br>50 µLIS<br>20 µL Perchloric acid 60%	LC/MSMS<br>C18, elution gradient methanol / buffer<br>6-TG : m/z 168-151	Results in pmol 6-TGN/ 0.2mL of whole blood, at hemoglobin content 120g/L

		300 µL 6mM DTT (4.5mM*)<br>100°C, 1h<br><br>3) 6-TGN – 6-MMP<br>0-10000 pmol /0.2ml whole blood<br>IS : 6-TG$^{13}C_2{}^{15}N$<br>6-MMPd_3	6-MMP : m/z 167-152<br><br>Extraction efficiency<br>6TG: 60%, 6-MMPN: 100%	Comparison with Lennard 1992 and Dervieux - Boulieu 1998
Rosdiana 2018 [44]	EDTA<br>WP RBC	**1)Final volume : 1800 µL**<br>400 µL RBC<br>800 µL IS,<br>600 µL NH_4OH/ acetonitrile<br>2) Evaporation at 55¨C<br>Reconstitution NH_4OH and Injection<br>3) 6-TGN: 9.9 -1979<br>6-MMP 10-2000 ng/mL<br>IS : bisoprostol	LC/MSMS<br>Acquity HSS T3 column (40°C)<br>Isocratic elution : Acetonitril-formic acid / H2O – formic acid<br><br>6TG : m/z 168-151<br>6-MMP : m/z 167-152<br>Bisoprostol : m/z : 326-116	Results in pmolx8.10^8 RBC
Yoo 2018 [45]	EDTA<br>WP RBC	**1) Final volume : 320 µL**<br>50 µL RBC<br>200 µL 0.2M DTT (*125 mM)<br>30 µL IS<br>40 µL Perchloric acid 70%<br>1h, 100°C<br><br>3)6-TGN : 0.1–10 µM<br>6-MMP : 0.5-100	LC/MSMS<br>6-TGN m/z 168.0→150.9<br>6-MMP m/z 158.0→110.0<br>6-TG^{13}C2^{15}N m/z 171.0→154.1<br>6-MMP-d3 m/z 161.1→110.1<br><br>Extraction efficiency<br>6-TGN: 71%, 6-MMPN: 96.4%	

		IS : 6-TG-$^{13}C2^{15}N$<br>16-MMP-d_3		

ACN: acetonitrile, 5-BU: Bromouracil, DTT: dithiothreithol - PM: 154, DTT * : DTT final sample concentration, EDTA: Ethylene-diamine-tetraacetic acid, HBSS: Hanks-balanced salt solution, Li-H: Lithium heparinate, PBS: phosphate buffered saline, PMA: phenylmercury acetate, SMX: sulfamethoxazole, W-P RBC: washed_packed RBC, saline: 0.9% sodium chloride, ESI-ESI-MS-MS: ion-exchange liquid chromatography with tandem mass spectrometry, 6TIA :6-thioxanthine riboside-5'-phosphate, TG : thioguanine , MTG : methylthioguanine, MMP : methylmercaptopurine, 8-bromoadenine, RT : retention time

Table S7 . Review of sample stability data during pre-analytical and analytical methods to quantify 6-TGN and 6-MMPN in red blood cells.

References	Compounds measured Analytical method	Stability information
Lennard 1987 [27]	6-TGN HPLC-UV	Extracted thiopurines (from acid hydrolysis) : stables in 0.1M hydrochloric acid for 36h at room temperature (autosampler) or 7 days at 4°C
Dervieux 1998 [28]	6-TGN, 6-MMPN	Formation of a Me6-MPN derivate during acid hydrolysis<br>No effect on 6-TGN and Me6-MPN derivates kept 24h at room temperature after the hydrolysis step
Pike 2001 [38] *Adapted from Erdman*[49]	6-TGN, 6-MMPN HPLC fluorescence	No difference in 6-TGN and 6-MMPN concentrations in whole blood and washed RBC<br>Whole blood samples kept at room temperature 1) for 7 days: 6-TGN : 2-4% reduction each day, 2) for 24 weeks : 12% 6-MMPN : no significant change<br>Whole blood samples kept at -80°C : 6-TGN 1) 1% reduction after 1 week, 2) 12% reduction after 24 weeks. 6-MMPN: No change in 24 weeks
De Graaf 2008 and 2010 [52-53]	6-TGN 6-MMPN HPLC- UV	Stability tests: 7 days at room temperature : 6-TGN decrease to 53% and 6-MMP to 55%<br>7 days at 4°C : 6-TGN decrease to 90% and 6-MMPN to 86%<br>6 months at -20°C : 6-TGN decrease to 90%, 6-MMPN decrease to 80%<br>6 months at -80°C : 6-TGN no decrease, 6-MMP decrease to 80%
Kirchherr 2013 [43]	6-TGN 6-MMPN	Stability / recovery of extracted samples kept 24h at 14°C (autosampler) : 103 (6.7)% for 6-TGN and 99 (3.9%) for 6-MMPN
Rosdiana 2018 [44]	6-TGN 6-MMPN LC/MSMS	Working solutions of 6-TGN and 6-MMPN in 0.5% NH4OH : stable when kept 1) at room temperature for 6 hours, 2) at 2°-8°C for 7 days<br>Working solutions of 6TGN and 6-MMP in RBCs 1) kept at room temperature for 18 hours : over 15% reduction of concentrations for both compounds, 2) stable kept at _20°C for 112 days:
Pecher 2018 [45]	12 Thiopurines metabolites IEC-EIC-MS/MS	6-TGN and 6-MMPN with DTT(10mM), EDTA and PB (50 mM, pH 7.4) stable when kept 4°C for 4h and for 5 days (within 95-105% of expected values.<br>Better results if washing and dilution in PBS compared to 0.9% NaCl
Yoo 2018 [46]	6-TGN 6-MMPN	6-TGN and 6-MMPN concentrations are stable at 25°C and 4°C for 4 hours 6-TGN decreases by 5% when stored at –70°C for 180 days and by 30% when stored at –20°C for 180 days<br>6-MMPN decreases by 10% at both temperatures<br>6-TGN decreased by more than 20% and 6-MMPN by more than 40% in whole blood samples kept at 25°C for 2 days

		6-TGN decreased by 20% in whole blood samples at 4°C on day 4.
Present report	6-TGN<br>6-MMPN<br>HPLC- UV	There was limited impact on 6-TGN and 6-MMPN concentrations if 1) washed and resuspended RBC are kept at -20° for 15 days or are submitted at 3 freeze/thaw cycles, 2) if extracted samples are left at room temperature for 48 to 72 hours, or kept at -40°C over 6 months,

4 TPN: thiopurine nucleotides, PB : phosphate buffer

Figure S1

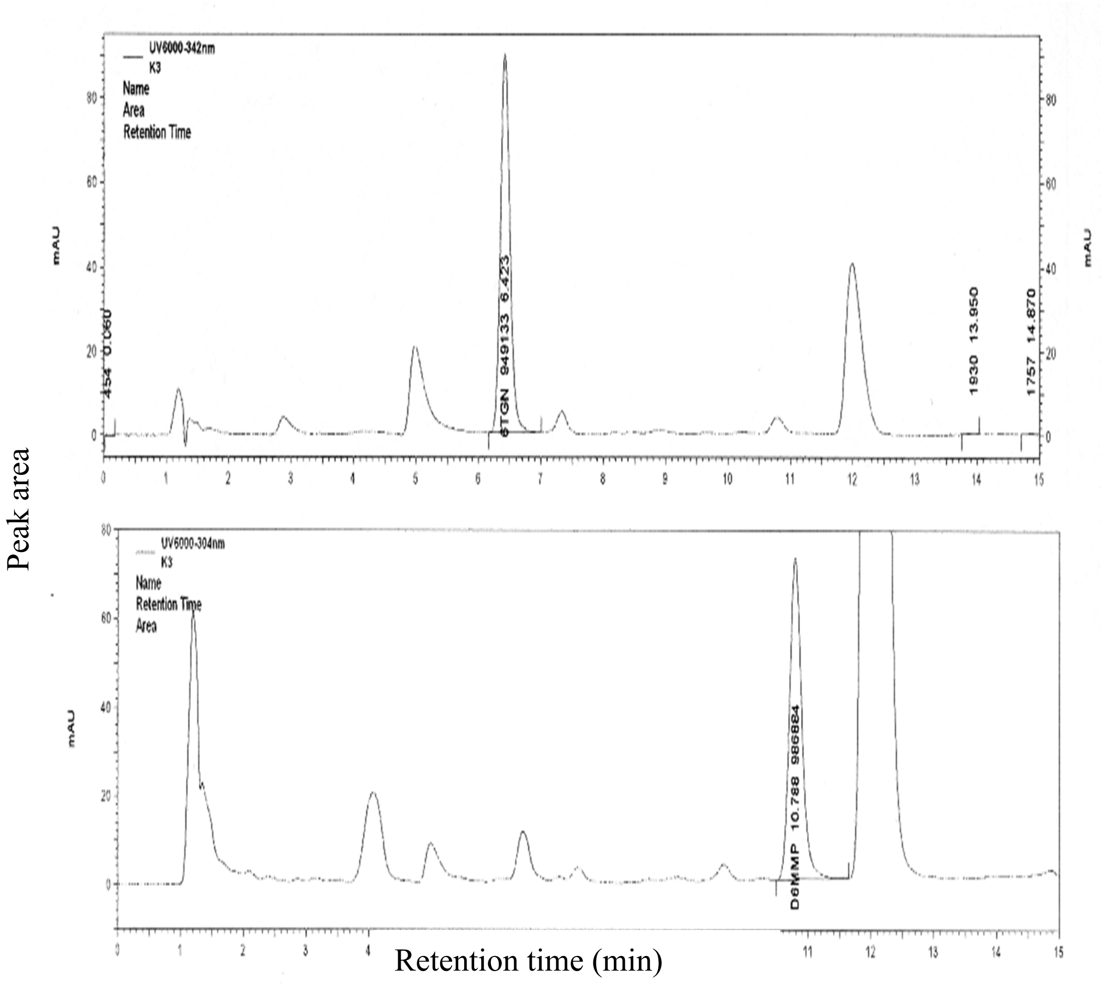

HPLC typical chromatograms showing 6-TGN and 6-MMP(d) peaks at the 342 and 304 nm
